# Supplementary material for: The causal role of circulating inflammatory markers in osteoporosis: a bidirectional Mendelian randomized study
Source: Front Immunol. 2024 Jul 18;15:1412298. doi: 10.3389/fimmu.2024.1412298 (PMC11291241; doi:10.3389/fimmu.2024.1412298)
Supplement: Supplementary file 1 [file DataSheet_1.zip › supplementary material/CIM-OSTEOPOROSIS /GCST90274814.csv_leave_one_out.pdf]

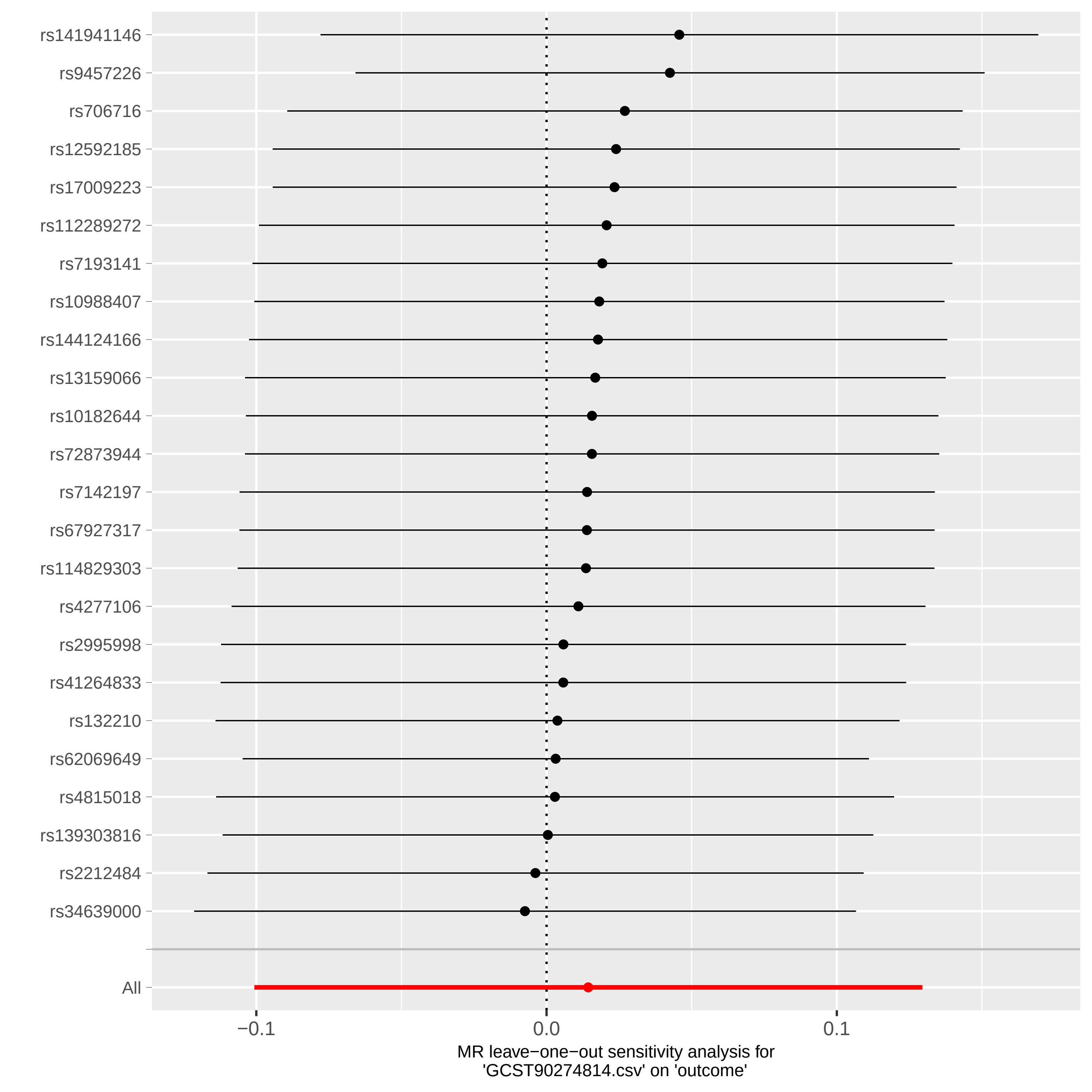
rs141941146
rs9457226
rs706716
rs12592185
rs17009223
rs112289272
rs7193141
rs10988407
rs144124166
rs13159066
rs10182644
rs72873944
rs7142197
rs67927317
rs114829303
rs4277106
rs2995998
rs41264833
rs132210
rs62069649
rs4815018
rs139303816
rs2212484
rs34639000
All
−0.1
0.0
0.1
MR leave−one−out sensitivity analysis for
'GCST90274814.csv' on 'outcome'
